# Influence of dose and route of administration on the outcome of infection with the virulent *Neospora caninum* isolate Nc-Spain7 in pregnant sheep at mid-gestation

**DOI:** 10.1186/s13567-018-0539-5

**Published:** 2018-05-08

**Authors:** Roberto Sánchez-Sánchez, Ignacio Ferre, Michela Re, Javier Regidor-Cerrillo, Javier Blanco-Murcia, Luis Miguel Ferrer, Teresa Navarro, Manuel Pizarro Díaz, Marta González-Huecas, Enrique Tabanera, Julio Benavides, Luis Miguel Ortega-Mora

**Affiliations:** 1 SALUVET, Animal Health Department, Faculty of Veterinary Sciences, Complutense University of Madrid, Ciudad Universitaria s/n, 28040 Madrid, Spain; 2 Department of Animal Medicine and Surgery, Faculty of Veterinary Sciences, Complutense University of Madrid, Ciudad Universitaria s/n, 28040 Madrid, Spain; 3 Departamento de Patología Animal, Facultad de Veterinaria, C/Miguel Servet 177, 50013 Zaragoza, Spain; 4 Livestock Health and Production Institute (ULE-CSIC), 24346 León, Spain

## Abstract

Experimental infections in pregnant sheep have been focused on studying the effect of the time of challenge on the outcome of *N. caninum* infection, whereas the impact of the dose and route of challenge has not been studied in depth. Therefore, clinical outcome, immune responses, parasite detection and burden, and lesion severity in placental tissues and foetal brains were investigated in 90-day-pregnant sheep inoculated intravenously with 10^5^ (G1), 10^4^ (G2), 10^3^ (G3), or 10^2^ (G4) tachyzoites or subcutaneously with 10^4^ (G5) tachyzoites of the virulent Nc-Spain7 isolate and an uninfected group (G6). Comparing challenge doses, G1 was the only group that had 100% abortion. Likewise, IFNγ levels in G1 increased earlier than those in other intravenously infected groups, and IgG levels on day 21 post-infection (pi) were higher in G1 than those in other intravenously infected groups. Concerning vertical transmission, G1 shows a higher parasite burden in the foetal brain than did G2 and G3. Comparing routes of administration, no differences in foetal survival rate or parasite load in the foetal brain were found. Although G2 had higher IFNγ levels than G5 on day 10 pi, no differences were found in humoral immune responses. Because the outcome after intravenous infection with 10^5^ tachyzoites was similar to that observed after intravenous infection with 10^6^ tachyzoites used in a previous work (100% abortion and vertical transmission), we conclude that it may be reasonable to use 10^5^ tachyzoites administered by the intravenous route in further experiments when assessing drugs or vaccine candidates.

## Introduction

*Neospora caninum* is an obligate intracellular apicomplexan parasite considered one of the leading infectious causes of abortion in cattle worldwide [1–3]. Recent studies suggest that *N. caninum* could also be a relevant abortifacient in some small ruminant management systems [4] or even the main cause of reproductive losses in some flocks [5, 6]. The pathogenesis of ovine neosporosis is poorly understood and, in contrast to the clinical outcome in cattle, infection during mid-pregnancy in sheep results in severe clinical outcome, since most of the animals abort or, less frequently, produce weak lambs [7, 8].

In pregnant sheep, infective doses of 10^7^–10^8^ tachyzoites results in a high percentage of abortions [9–12]. In a study comparing different infective doses, a strong relationship between the challenge dose of Nc-NZ1, Nc-NZ2 and Nc-NZ3 *N. caninum* tachyzoites and the clinical outcome was found in pregnant sheep at mid-gestation [9]. To date, there are no studies comparing the outcome of *N. caninum* experimental infection using different routes of inoculation in pregnant sheep, although, in cattle, this is crucial because intravenous inoculation is associated with a more severe clinical presentation than subcutaneous inoculation [13]. Likewise, there are clear differences concerning the outcome of the infection among parasite isolates [14]. The Nc-Spain7 isolate [15] is a very well-characterized virulent isolate tested so far in three experimental ruminant models, sheep [8], goats [16] and cattle [17–19]. Recently, the Nc-Spain7 isolate has been evaluated at different times during gestation in pregnant sheep, suggesting that the time of infection plays a key role in the pathogenesis of the disease [8].

Therefore, the aim of this study was to investigate the effect of challenge dose and route of administration on the outcome of experimental infection in ewes at mid-term gestation using the Nc-Spain7 isolate based on the clinical course of disease, cellular and humoral immune responses, lesion development and parasite detection and burden in placental and foetal tissues. This experiment allowed the refinement and standardization of an exogenous transplacental transmission model for ovine neosporosis.

## Materials and methods

### Animals and experimental design

Forty Rasa Aragonesa breed female lambs aged 3 months were selected from a commercial flock after checking their seronegativity for *T. gondii*, *N. caninum*, Border disease virus (BDV), Schmallenberg virus (SBV), *Coxiella burnetii* and *Chlamydia abortus* by ELISA. Animals were maintained in isolation at Zaragoza University (Spain) facilities and at 12 months old were oestrus synchronised by insertion of intravaginal progestogen-impregnated sponges (Chronogest^®^ 20 mg fluorogestone acetate, MSD Animal Health, Salamanca, Spain) for 14 days. At the time of removal, 480 UI of pregnant mare serum gonadotrophin (PMSG) (Foligon^®^ 6000 UI, MSD Animal Health, Salamanca, Spain) was administered to each ewe through intramuscular injection as previously described [20]. After 48 h, ewes were mated with Rasa Aragonesa breed tups for 2 days, after which, the rams were removed from the ewes. Pregnancy and foetal viability were confirmed by ultrasound scanning (US) on day 40 after mating, and twenty-seven pregnant sheep were selected for the experiment. Pregnant ewes (*n* = 27) were randomly distributed into six experimental groups at Clinical Veterinary Hospital facilities of Complutense University of Madrid (Spain). Twenty-four ewes were inoculated intravenously into the jugular vein at 90 days of gestation (dg) with 10^5^ (group 1, G1; *n* = 6), 10^4^ (group 2, G2; *n* = 5), 10^3^ (group 3, G3; *n* = 5), 10^2^ (group 4, G4; *n* = 4) tachyzoites; or subcutaneously over the left prefemoral lymph node with 10^4^ tachyzoites (group 5, G5; *n* = 4) of the Nc-Spain7 bovine isolate [15]. The three remaining pregnant ewes were allocated to group 6 (G6; *n* = 3), acted as uninfected controls and received an intravenous inoculum of phosphate-buffered saline (PBS) at 90 dg (Table 1).

**Table 1:** **Experimental design**

Group	Number of pregnant ewes	Number of foetuses	Ratio foetuses/dam	Inoculum	Route of inoculation
G1	6	12	2	Nc-Spain7 10^5^ tachyzoites	IV
G2	5	11	2.20	Nc-Spain7 10^4^ tachyzoites	IV
G3	5	13	2.60	Nc-Spain7 10^3^ tachyzoites	IV
G4	4	7	1.75	Nc-Spain7 10^2^ tachyzoites	IV
G5	4	8	2	Nc-Spain7 10^4^ tachyzoites	SC
G6	3	5	1.66	PBS	IV

*IV* intravenous route, *SC* subcutaneous route

### Parasite culture and dose preparation

Tachyzoites of the Nc-Spain7 isolate were routinely maintained in cultured MARC-145 cells as described previously [21]. For the challenge, tachyzoites (passage 19) were recovered from culture flasks when they were still largely intracellular (> 80% of undisrupted parasitophorous vacuoles), and infected cells were repeatedly passed through a 25-gauge needle at 4 °C. The number of viable tachyzoites was determined by Trypan blue exclusion (typically 95–99%) followed by counting the viable tachyzoites in a Neubauer chamber. Subsequently, the concentration of viable tachyzoites was adjusted to the required dose (10^5^, 10^4^, 10^3^ and 10^2^) by dilution in PBS in a final volume of 1 mL. Tachyzoites were administered to pregnant ewes within 30 min of harvesting from cell culture.

### Clinical monitoring and collection of samples

Pregnant ewes were observed daily throughout the entire experimental period. Rectal temperatures were recorded daily from day 0 until 14 days pi and then weekly. Animals were considered febrile when the rectal temperature was over 40 °C [22].

In G5, which was subcutaneously inoculated with Nc-Spain7 tachyzoites, changes in the left prefemoral lymph node compared to the right prefemoral lymph node by palpation were recorded daily until its resolution. The left prefemoral lymph node was regarded as enlarged if its size exceeded that of the right prefemoral lymph node by at least 50%.

Blood samples to evaluate immune responses were collected before infection, on days 3, 5, 7 and 10 pi and then weekly by jugular venipuncture into 5 mL vacutainer tubes (Becton–Dickinson and Company, Plymouth, UK) with lithium heparin as anticoagulant and without anticoagulant. Tubes without anticoagulant were allowed to clot and centrifuged to obtain serum, and samples were stored at − 80 °C until analysis.

Foetal viability was assessed by transabdominal ultrasonography (US) to monitor foetal heartbeat and movements once weekly for the first 2 weeks post-infection (pi) and then twice weekly until detection of foetal death. When foetal death was detected in any of the foetuses or 24 h after parturition, dams and lambs were first sedated with xylazine (Rompun, Bayer, Mannhein, Germany) and then immediately euthanized by an IV overdose of embutramide and mebezonium iodide (T61, Intervet, Salamanca, Spain).

At necropsy, six randomly selected placentomes were recovered from each placenta of aborted dams, were transversally cut into slices of 2–3 mm thickness and were stored in 10% formalin for histopathological examinations. The rest of the placentomes were stored at − 80 °C for further parasite DNA detection by PCR. In dams that gave birth, six randomly selected cotyledons were recovered and stored at − 80 °C for further parasite DNA detection by PCR. From foetuses, the foetal brain was stored at − 80 °C for DNA extraction and fixation in 10% formalin. Foetal thoracic and abdominal fluids or precolostral serum were also collected from aborted foetuses or newborn lambs, respectively, and maintained at − 80 °C for serology.

Lambs were weighed and sampled for blood at birth and were euthanized 24 h after birth. To avoid any accidental suckling from lambs born overnight, udders were covered with a piece of cloth 1 week before the expected date of delivery as a preventive measure.

### Peripheral blood stimulation assay and interferon-gamma (IFNγ) production analysis

Peripheral blood stimulation assay was carried out and interferon-gamma (IFNγ) production was evaluated as previously described [23]. Briefly, heparinised blood was cultured in 24-well flat-bottom plates in the presence of either soluble *N. caninum* antigens or concanavalin A (ConA, Sigma-Aldrich, Madrid, Spain), both at final concentrations of 5 μg/mL. Plates were incubated in a 5% CO_2_/37 °C/100% humidity atmosphere for 24 h. They were then centrifuged at 1000×*g* for 10 min at 4 °C, and culture supernatants were assayed for IFN-γ detection using a commercial bovine enzyme immunoassay kit with a capture monoclonal antibody (MT17.1) showing cross-reactivity with ovine IFNγ (Mabtech AB, Sweden) as previously described [8].

### Serological analyses: ELISA and IFAT

*Neospora caninum*-specific IgG antibody levels were measured using an in-house indirect enzyme-linked immunosorbent assay (ELISA) as previously described [23]. Briefly, 96-well microtiter plates (Thermo Fisher Scientific, Waltham, USA) were coated with 100 µL soluble *N. caninum* antigen (1 µg/mL in 100 mM carbonate buffer pH 9.6) overnight at 4 °C [24]. Plates were blocked and serum samples were diluted 1:100 using 3% bovine serum albumin diluted in PBS containing 0.05% Tween 20 (PBS-T). Subsequently, horseradish peroxidase-conjugated protein G (Sigma-Aldrich, Madrid, Spain) diluted 1:2000 in PBS-T was added and after that, ABTS (Roche, Basilea, Switzerland) was used as substrate. The reaction was stopped by 0.3 M oxalic acid and the optical density (OD) was read at 405 nm (OD405). For each plate, values of the OD were converted into a relative index percent (RIPC) using the following formula: RIPC = (OD405 sample—OD405 negative control)/(OD405 positive control–OD405 negative control) × 100. A RIPC value ≥ 10 indicates a positive result.

An indirect fluorescent antibody test (IFAT) was used to detect specific IgG anti-*Neospora* antibodies in foetal fluids and precolostral sera, according to the technique described by [24] and used in previous studies [23].

### Histopathology and lesion scoring

After fixation in formalin for 5 days, formalin-fixed samples were cut, embedded in paraffin wax, and processed by standard procedures for haematoxylin and eosin (HE) staining. Conventional histological evaluation was carried out on all the sections. The analysis was based on the observation of lesions according to previous descriptions [8], and the lesions were classified as none detected/unrelated (−), mild lesions (+) (in the placentome, 0–33.3% of the placentome show lesions; in the foetal brain, presence of local encephalitis), moderate lesions (++) (in the placentome, 33.3–66.6% of the placentome show lesions; in the foetal brain, presence of diffuse encephalitis) and severe lesions of ovine neosporosis (+++) (in the placentome, 66.6–99.9% of the placentome show lesions; in the foetal brain, presence of diffuse encephalitis and necrosis).

### DNA extraction and PCR for parasite detection and quantification in tissues

Genomic DNA was extracted from 50 to 100 mg of maternal and foetal tissue samples using the commercial Maxwell^®^ 16 Mouse Tail DNA Purification Kit, developed for the automated Maxwell^®^ 16 System (Promega, Wisconsin, USA), according to the manufacturer’s recommendations. The concentration of DNA for all samples was determined by spectrophotometry and adjusted to 50–100 ng/μL.

PCR was carried out on six placentome samples from aborted dams or cotyledons in dams that gave birth and three foetal brain samples. Parasite DNA detection was carried out by nested PCR adapted to a single tube as previously described [18, 25]. Each reaction was performed in a final volume of 25 μL with 5 μL of sample DNA. Samples from the uninfected group (G6) were included in each round of DNA extraction and PCR as negative controls. Positive PCR controls with *N. caninum* genomic DNA equivalent to 10, 1 and 0.1 tachyzoites in 100 ng of sheep DNA were also included in each batch of amplifications. Ten microlitre aliquots of the PCR products were visualized under UV light in a 1.5% agarose/ethidium bromide gel to detect the *N. caninum*-specific 247 bp amplification product.

Placenta and foetal brain samples that tested positive by nested-PCR were adjusted to 20 ng DNA/μL, and the parasite load was quantified using real-time PCR. Primer pairs from the *N. caninum* Nc-5 sequence [26] were used for parasite quantification, and primers from the β-actin gene [27] were used for the quantification of host DNA. Amplification reactions were performed as described by [23].

### Statistical analysis

The occurrence of foetal death was analysed by the Kaplan–Meier survival method. Foetal survival curves were then compared by the Log-rank (Mantel–Cox) test, and the median foetal survival time, i.e., the day at which 50% of the foetuses aborted, was calculated. Weight of the lambs and antibody responses in foetuses and lambs were compared using the non-parametric Kruskal–Wallis test followed by the Dunn’s test for comparisons between groups and the Mann–Whitney test for pairwise comparisons. Rectal temperatures were analysed using a two-way ANOVA of repeated measures test until 14 days pi and a one-way ANOVA test afterwards. Humoral and cellular immune responses for each experimental group were analysed using a two-way ANOVA of repeated measures test until 28 days pi and a one-way ANOVA test afterwards. However, cellular immune responses in G1 were analysed using one-way ANOVA test. Differences in PCR detection of parasite DNA were evaluated using the χ^2^ or Fisher Exact F-test. Differences in parasite burdens and lesion severity were analysed using the non-parametric Kruskal–Wallis test followed by Dunn’s test for comparisons between groups and the Mann–Whitney test for pairwise comparisons. Statistical significance for all analyses was established at *P* < 0.05. Differences that show *P* values ≥ 0.05 and ≤ 0.18 were considered to be trending towards statistical signification. All statistical analyses were carried out using GraphPad Prism 6.01 software (San Diego, CA, USA).

## Results

### Clinical observations

**Figure 1:**
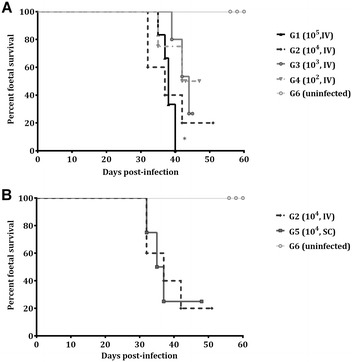
**Foetal survival curves.** Foetal survival curves from intravenously challenged animals (**A**) and from IV and SC challenged animals with 10^4^ Nc-Spain7 tachyzoites (**B**). Each point represents the percentage of surviving animals on that day, and downward steps correspond to observed deaths. Foetal survival curves were compared by the log-rank (Mantel–Cox) test. For significant differences between foetal survival curves of infected groups, (*) indicates *P* < 0.05.

Foetal death was detected from 32 to 44 days pi by US in 6 out of 6 pregnant ewes in G1 (median abortion day 38), 4 out of 5 pregnant ewes in G2 (median abortion day 34), 3 out of 5 pregnant ewes in G3 (median abortion day 42), 2 out of 4 pregnant ewes in G4 (median abortion day 38) and 3 out of 4 pregnant ewes in G5 (median abortion day 35) (Table 2). The median survival times were 38, 37, 44, 51 and 36 days for G1, G2, G3, G4 and G5, respectively (Figure 1). All aborted dams in G3 and G4, one aborted dam in G1 and one aborted dam in G5 had twin pregnancies, and they showed, before being euthanized, foetal death in one of the foetuses and foetal heartbeat and movements by US in the other. One mummified foetus in G1 and three mummified foetuses in G2 were found at necropsy (Table 2). All dams from the uninfected group (G6) gave birth, and a significant difference was found in the foetal survival rate compared to that in G1 (*P* < 0.05). Regarding challenge doses, compared to G1, a higher foetal survival rate was found in G3 (*P* < 0.05), and in G4, the statistical difference (*P* = 0.06) shows a trend towards significance (Figure 1A). Regarding routes of administration, no significant differences in the foetal survival rate between G2 and G5 were found (Figure 1B). Non-aborted dams in G3, G4 and G5 gave birth prematurely between days 132 and 141 of pregnancy. Stillborn lambs and lambs exhibiting weakness, recumbency and unresponsiveness to external stimuli and dying within 24 h after birth were found in 7 of 7 lambs in G3 and 2 of 3 lambs in G4 and G5. In contrast, non-aborting dams in G2 and dams in G6 gave birth to healthy lambs between days 144 and 150 of pregnancy. A significant decrease in the weight of the lambs from G3 (1511.5 ± 85.5 g) (*P* < 0.01), G4 (2429 ± 447.4 g) (*P* < 0.05) and G5 (2213.3 ± 528.7 g) (*P* < 0.05) was found compared to that in G6 (4037.4 ± 354.7 g), whereas no statistical analysis was performed in G2 since only one lamb was born (2919 g).

**Table 2:** **Detection of parasite DNA, parasite load and histopathological changes in foetal brains and placental tissues from sheep and serological analysis of the foetuses after challenge infection with the Nc-Spain7 isolate at mid-gestation**

Group	Animal id.	Foetal death (days pi)^a^	Placentomes/cotyledons^b^			Foetal brain															
						Foetus/lamb 1				Foetus/lamb 2				Foetus/lamb 3				Foetus/lamb 4			
			HP^c^	DNA^e^	qPCR^f^	IFAT^d^	HP^c^	DNA^e^	qPCR^f^	IFAT^d^	HP^c^	DNA^e^	qPCR^f^	IFAT^d^	HP^c^	DNA^e^	qPCR^f^	IFAT^d^	HP^c^	DNA^e^	qPCR^f^
Group 1 (10^5^ tachyzoites, IV)	1.1	38	+++	6/6	2030.18 ± 2021.53	1:32	+++	3/3	47.53 ± 22.41	1:256	*	0/3	0.01								
	1.2	40	+++	6/6	1990.74 ± 543.90	1:256	++	2/3	3.40 ± 3.01												
	1.3	40	++	6/6	3355.17 ± 2407.07	1:1024	+++	3/3	8.47 ± 2.67												
	1.4	37	++	6/6	2028.50 ± 2434.11	1:128	++	2/3	14.42 ± 13.01	1:256	+++	3/3	584.97 ± 403.74	1:32	*	3/3	97.72 ± 109.14	^β^	^β^	^β^	^β^
	1.5	35	+++	6/6	3296.38 ± 1231.41	1:64	+++	3/3	141.14 ± 69.34	1:32	*	3/3	56.18 ± 27.50								
	1.6	38	+++	6/6	7974.29 ± 4660.05	1:1024	++	2/3	13.26 ± 15.57	1:1024	++	3/3	16.86 ± 10.67								
Group 2 (10^4^ tachyzoites, IV)	2.1	32	+++	6/6	7420.68 ± 6570.82	1:256	++	3/3	13.38 ± 6.04												
	2.2		NA	4/4^γ^	3992.48 ± 1562.24^γ^	1:800	+	2/3	5.16 ± 5.59	^β^	^β^	^β^	^β^	^β^	^β^	^β^	^β^				
	2.3	32	+++	6/6	4548.31 ± 4733.95	1:128	+	3/3	19.61 ± 12.04												
	2.4	37	+	6/6	1930.36 ± 2034.10	1:256	++	2/3	5.54 ± 6.06	^β^	^β^	^β^	^β^								
	2.5	42	+++	6/6	6829.41 ± 3917.31	1:64	+	3/3	2.70 ± 0.86	1:64	++	3/3	20.29 ± 8.74	NA	++	3/3	11.97 ± 10.24	1:128	++	3/3	3.31 ± 2.49
Group 3 (10^3^ tachyzoites, IV)	3.1	39	++	6/6	12 260.49 ± 9266.82	1:32	++	3/3	9. 48 ± 5.47	1:256	++	3/3	23.56 ± 13.52								
	3.2	42	++	6/6	7370.42 ± 9178.25	1:256	++	3/3	2.27 ± 0.73	1:512	++	0/3	0.01								
	3.3	44	++	6/6	2307.22 ± 1754.57	1:256	++	3/3	39.60 ± 43.86	1:128	++	0/3	0.01								
	3.4		NA	5/6	62.07 ± 95.88	1:400	++	2/3	3.07 ± 2.72	1:400	++	2/3	3.33 ± 3.52	1:200	+	0/3	0.01	1:256	+	1/3	0.67 ± 1.15
	3.5		NA	6/6	823.41 ± 714.63	1:1024	++ ^α^	3/3	7.74 ± 3.20	1:256	+	3/3	6.54 ± 0.30	1:64	++	3/3	15.91 ± 7.92				
Group 4 (10^2^ tachyzoites, IV)	4.1		NA	6/6	1334.88 ± 561.06^γ^	1:400	+	3/3	12.44 ± 7.18	1:64	*	3/3	37.24 ± 17.02								
	4.2		NA	6/6	6160.29 ± 4646.09^γ^	1:400	+	1/3	1.22 ± 2.11												
	4.3	35	++	6/6	2101.24 ± 1290.86^γ^	1:512	++	3/3	23.03 ± 3.12	1:1024	++	3/3	14.35 ± 12.77								
	4.4	42	++	6/6	3226.22 ± 2350.13^γ^	1:512	++	3/3	45.58 ± 22.09	1:128	++	3/3	20.07 ± 13.80								
Group 5 (10^4^ tachyzoites, SC)	5.1		NA	6/6	65.40 ± 27.49^γ^	1:800	+	3/3	3.87 ± 1.85	1:200	+	1/3	0.65 ± 1.12	1:800	+	3/3	7.22 ± 5.87				
	5.2	35	+	6/6	707.20 ± 614.80	1:512	++	3/3	16.07 ± 2.49	1:256	++ ^α^	2/3	2.62 ± 2.31								
	5.3	32	+++	6/6	38 294.17 ± 23 816.82	1:512	+++	3/3	114.35 ± 34.34												
	5.4	37	+	6/6	781.48 ± 444.87^γ^	1:128	+	3/3	6.32 ± 5.64	1:256	++	3/3	2.81 ± 1.58								
Group 6 (uninfected)	6.1		NA	0/6	0.01	–	–	0/3	0.01	–	–	0/3	0.01								
	6.2		NA	0/6	0.01	–	–	0/3	0.01	–	–	0/3	0.01								
	6.3		NA	0/6	0.01	–	–	0/3	0.01												

^a^Day post-challenge when foetal death was detected by ultrasonography. The remaining foetuses lived until the end of the experiment

^b^Placentomes in ewes that aborted and cotyledons in ewes that gave birth; “NA” samples from cotyledons were not evaluated for histopathology

^c^Histopathological lesion severity: none detected/unrelated (−), mild lesions (+) (in placentome, 0–33.3% of the placentome show lesions; in foetal brain, presence of local encephalitis), moderate lesions (++) (in placentome, 33.3–66.6% of the placentome show lesions; in foetal brain, presence of diffuse encephalitis) and severe lesions of ovine neosporosis (+++) (in placentome, 66.6–99.9% of the placentome show lesions; in foetal brain, presence of diffuse encephalitis and necrosis). * Autolysed; ^α^ No histopathological changes were found in one of the slides analysed

^d^IFAT IgG antibody titres in foetal body fluids and in precolostral serum collected after birth in lambs born alive; “NA” not available

^e^Fractions represent the number of positive samples by nested PCR/number of samples examined

^f^Mean parasite load (tachyzoites/mg tissue) and standard deviation (SD). Considering that the *N. caninum* detection limit by real-time PCR is 0.1 parasites, negative samples (0 parasites) were represented as 0.01

^β^Mummified foetuses were not evaluated for any of the parameters

^γ^Samples from placental tissues exhibiting DNA degradation were excluded from parasite detection and/or parasite load analysis

**Figure 2:**
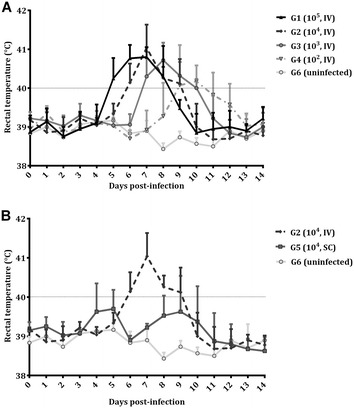
**Rectal temperatures after** ***N. caninum*** **challenge in pregnant ewes. A** Rectal temperatures in intravenously challenged animals (**A**) and in IV and SC challenged animals with 10^4^ Nc-Spain7 tachyzoites (**B**). Each point represents the mean + SD at different times for each group. Rectal temperatures represented in figure were analysed using two-way ANOVA of repeated measures.

Concerning rectal temperatures, all intravenously challenged animals had fever (rectal temperature above 40 °C) at some time point until day 14 pi. Compared to the uninfected group (G6), a significant increase in rectal temperature was found from day 5 pi (*P* < 0.01) to day 8 pi (*P* < 0.0001) in G1, from day 6 pi (*P* < 0.001) to day 9 pi (*P* < 0.001) in G2, from day 7 pi (*P* < 0.001) to day 10 pi (*P* < 0.0001) in G3 and from day 9 pi (*P* < 0.001) to day 11 pi (*P* < 0.001) in G4, with maximum mean rectal temperature on day 7 pi in G1 and G2, on day 8 pi in G3 and on day 10 pi in G4 (Figure 2A). Likewise, in the subcutaneously challenged group (G5), a significant increase in rectal temperature on day 8 pi (*P* < 0.05) was found when compared to that in the uninfected group (G6); moreover, significant increases in rectal temperatures on days 4 (*P* < 0.05), 5 (*P* < 0.05) and 9 pi (*P* < 0.05) were found compared to that on day 14 pi in G5. In contrast to the intravenously infected groups, no mean rectal temperature above 40 °C was found in the subcutaneously challenged group (G5) at any time, and only a few animals had fever (one ewe on days 4, 5, 8 and 10 pi and two ewes on day 9 pi) (Figure 2B). When comparing rectal temperatures between aborting ewes and ewes that gave birth, significant differences were found only in G4 since aborting ewes had higher rectal temperatures on days 8 (*P* < 0.05) and 9 pi (*P* < 0.001) than ewes that gave birth. Likewise, in G4, aborting ewes had fever on days 9 and 10 pi, whereas ewes that gave birth had fever on day 11 pi. The mean rectal temperature in the uninfected group (G6) remained below 39.5 °C throughout the monitoring period. From day 14 pi until the end of the experiment, no changes were found in rectal temperatures in the infected groups.

In G5, enlargement of the left prefemoral lymph node was observed in all pregnant sheep between days 2 and 14 pi.

### Cellular and humoral immune responses

**Figure 3:**
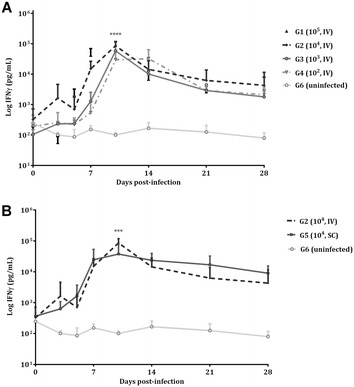
**Systemic IFNγ response after** ***N. caninum*** **challenge in pregnant ewes.** IFNγ response in intravenously challenged animals (**A**) and in IV and SC challenged animals with 10^4^ Nc-Spain7 tachyzoites (**B**). Each point represents the mean + SD at the different sampling times for each group. IFNγ levels from G1 on day 10 pi are not available due to problems in antigen stimulation assays of the peripheral blood. Data beyond day 28 pi are not included because several animals did not maintain pregnancy and were therefore sacrificed. Concentrations of IFNγ are expressed in pg/mL. The cellular immune response represented in figure was analysed using two-way ANOVA of repeated measures. For significant differences between infected groups, (***) indicates *P* < 0.001, and (****) indicates *P* < 0.0001.

IFNγ levels in supernatants recovered 24 h after *N. caninum* antigen stimulation are shown in Figure 3. IFNγ values increased on day 7 pi in G1 (*P* < 0.05) and on day 10 pi in G2 (*P* < 0.0001), G3 (*P* < 0.0001) and G5 (*P* < 0.05), whereas G4 did not show a significant increase in IFNγ levels at any time pi compared to the uninfected group (G6). Regarding challenge doses, on day 10 pi, G2 had higher IFNγ values than G4 (*P* < 0.0001) (Figure 3A). Regarding routes of administration, on day 10 pi, G2 had higher IFNγ response than G5 (*P* < 0.001) (Figure 3B). The ewe that gave birth in G2 had higher IFNγ levels on day 10 pi than those that aborted (*P* < 0.01), but ewes that gave birth in G3 had lower IFNγ levels than those that aborted (*P* < 0.001). In G4, aborting ewes had higher IFNγ response on day 10 pi than those that gave birth (*P* < 0.05); however, ewes that gave birth had higher IFNγ values on day 14 pi (*P* < 0.05) than those that aborted. No significant differences between ewes that gave birth and aborting ewes were found in G5. No significant differences between groups were found in IFNγ levels from days 14 to 28 pi. Likewise, all infected groups maintained low IFNγ levels from day 28 pi onwards (data not shown). None of the uninfected animals (G6) had IFNγ levels above basal levels recorded prior to inoculation throughout the experimental study.

**Figure 4:**
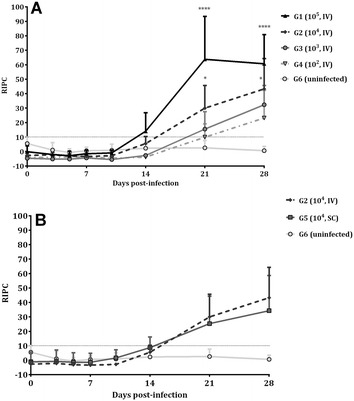
**Anti-*N. caninum*** **IgG response in serum.** IgG response in intravenously challenged animals (**A**) and in IV and SC challenged animals with 10^4^ Nc-Spain7 tachyzoites (**B**). Each point represents the mean + SD at the different sampling times for each group. Data beyond day 28 pi are not included because several animals did not maintain pregnancy and were therefore sacrificed. Serum levels of total IgG antibodies against *N. caninum* are expressed as a relative index percent (RIPC), according to the formula: RIPC = (OD405 sample − OD405 negative control)/(OD405 positive control − OD405 negative control) × 100. The humoral immune response shown in figure was analysed using two-way ANOVA of repeated measures. For significant differences between infected groups, (*) indicates *P* < 0.05 and (****) indicates *P* < 0.0001.

The *N. caninum*-specific IgG antibody response in dams is shown in Figure 4. When compared to those in the uninfected group (G6), IgG levels increased significantly from day 21 pi in G1 (*P* < 0.0001), G2 (*P* < 0.01) and G5 (*P* < 0.05) and from day 28 pi in G3 (*P* < 0.001) and G4 (*P* < 0.05). Moreover, when challenge doses were compared, G1 had higher IgG values on day 21 pi than all other intravenously infected groups (*P* < 0.0001) and on day 28 pi than G3 (*P* < 0.0001) and G4 (*P* < 0.0001). Likewise, G2 had higher IgG values than G4 on days 21 and 28 pi (*P* < 0.05) (Figure 4A). From day 28 pi until foetal death/birth occurred, G1 had higher IgG values than G2 (*P* < 0.01), G3 (*P* < 0.01) and G4 (*P* < 0.01) (data not shown). When routes of administration were compared, no significant differences in IgG serum levels were found between G2 and G5 at any time (Figure 4B). Likewise, no significant differences were found between aborting ewes and ewes that gave birth in any of the infected groups. Seroconversion in all animals from infected groups was observed at the time of abortion/birth. All uninfected animals (G6) had basal IgG levels throughout the experimental study.

The *Neospora*-specific IgG response in foetal fluids from aborted foetuses and stillborn lambs or precolostral sera collected from lambs is summarized in Table 2. Seropositive titres were detected in all aborted foetuses from infected groups, ranging from 1:32 to 1:1024. IFAT titre medians in aborted foetuses were 1:128 for G2, 1:256 for G1, G3 and G5, and 1:512 for G4 without significant differences among challenge doses or routes of administration. Precolostral sera yielded positive titres ranging from 1:200 to 1:800 in all lambs born from infected groups. IFAT titre medians were 1:800 in G2 and G5 and 1:400 in G3 and G4, without significant differences among challenge doses (no statistical analysis was performed in G2 because only one lamb was born). Specific IgG responses against parasite antigen were not detected in lambs from the uninfected group (G6).

### Pathology and lesion quantification

Placentas from aborting ewes show multifocal non-purulent necrotic placentitis consisting of foci of necrosis in the placentomes mainly located in the caruncular septa. Several of these foci show mineralization of the necrotic core area. No significant differences among challenge doses or routes of administration were found in the lesion severity in placentomes (Table 2).

A multifocal nonpurulent necrotizing encephalitis characterized by the presence of randomly distributed glial foci surrounded by mononuclear cells was observed in foetal brains from all infected groups. Concerning challenge doses, lesion severity in the foetal brain was higher in G1 compared to those in G2 (*P* < 0.05), G3 (*P* < 0.01) and G4 (*P* < 0.05). By contrast, concerning routes of administration, no differences in lesion severity in the foetal brain were found between G2 and G5 (Table 2). Although aborted foetuses in G1 had higher lesion severity in the foetal brain compared to those in G2 (*P* < 0.05), no significant differences in lesion severity in the foetal brain were found between lambs born in any of the infected groups or between aborted foetuses and lambs born in each group. No histopathological findings were found in the uninfected group (G6).

### Parasite detection and burden in placental tissues and foetal brain

**Figure 5:**
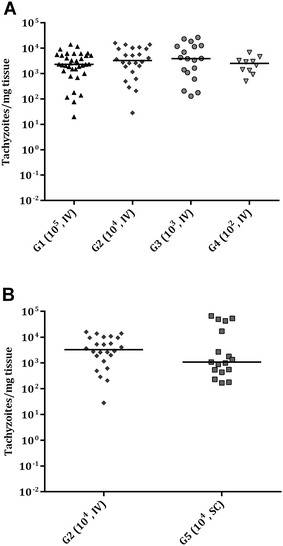
**Dot-plot graphs of** ***N. caninum*** **burdens in placentomes.** *N. caninum* burden in placentomes from intravenously challenged dams (**A**) and from IV and SC challenged animals with 10^4^ Nc-Spain7 tachyzoites (**B**). Each dot represents individual values of parasite burden (number of parasites per mg of host tissue), and medians are represented as horizontal lines. Considering that the *N. caninum* detection limit by real-time PCR is 0.1 parasites, negative samples (0 parasites) were represented on the log scale as < 0.1 (i.e., 10^−2^). Parasite burdens were analysed using the non-parametric Kruskal–Wallis test followed by the Dunn’s test for comparisons between groups, as well as the Mann–Whitney test for pairwise comparisons.

**Figure 6:**
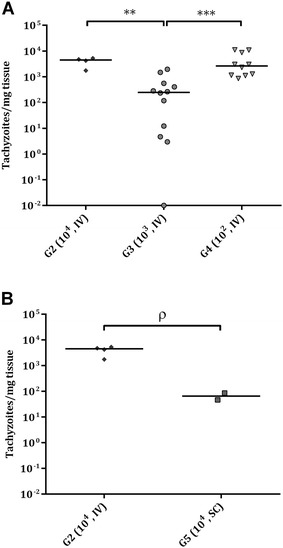
**Dot-plot graphs of** ***N. caninum*** **burdens in cotyledons.** *N. caninum* burden in cotyledons from intravenously challenged dams (**A**) and from IV and SC challenged animals with 10^4^ Nc-Spain7 tachyzoites (**B**). Each dot represents individual values of parasite burden (number of parasites per mg of host tissue), and medians are represented as horizontal lines. Considering that the *N. caninum* detection limit by real-time PCR is 0.1 parasites, negative samples (0 parasites) were represented on the log scale as < 0.1 (i.e., 10^−2^). Parasite burdens were analysed using the non-parametric Kruskal–Wallis test followed by the Dunn’s test for comparisons between groups, as well as the Mann–Whitney test for pairwise comparisons. (**) indicates *P* < 0.01, (***) *P* < 0.001, and (ρ) indicates a trend towards significant differences among infected groups in each tissue.

A few samples from placental tissues and foetal brain exhibited DNA degradation mainly due to mummification and were excluded from parasite detection and/or parasite load analysis (Table 2). *Neospora* DNA was detected in 100% of placentomes from ewes that aborted in G1 (36/36), G2 (24/24), G3 (18/18), G4 (12/12) and G5 (18/18), 100% of cotyledons from ewes that gave birth in G2 (4/4), G4 (12/12) and G5 (6/6) and 91.7% of cotyledons from ewes that gave birth in G3 (11/12) (Table 2). No significant differences in parasite detection in placentomes and cotyledons were found among challenge doses or between routes of administration. When analysing the mean parasite burden, measured as the number of tachyzoites per mg of tissue, no significant differences in parasite load in placentomes were found among challenge doses or between routes of administration (Figure 5A, B) (Table 2). Despite the low number of samples available, comparing challenge doses, parasite burden in cotyledons was significantly lower in G3 compared to those in G2 (*P *< 0.01) and G4 (*P *< 0.001) (Figure 6A). Likewise, comparing routes of administration, G2 had a higher parasite load in cotyledons, with a trend towards significance compared to G5 (*P *= 0.13) (Figure 6B) (Table 2).

**Figure 7:**
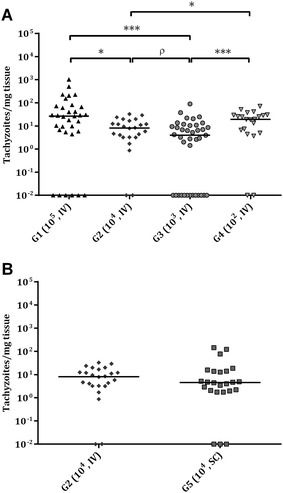
**Dot-plot graphs of** ***N. caninum*** **burdens in foetal brain.** *N. caninum* burden in foetal brain from intravenously challenged dams (**A**) and from IV and SC challenged animals with 10^4^ Nc-Spain7 tachyzoites (**B**). Each dot represents individual values of parasite burden (number of parasites per mg of host tissue), and medians are represented as horizontal lines. Considering that the *N. caninum* detection limit by real-time PCR is 0.1 parasites, negative samples (0 parasites) were represented on the log scale as < 0.1 (i.e., 10^−2^). Parasite burdens were analysed using the non-parametric Kruskal–Wallis test followed by the Dunn’s test for comparisons between groups, as well as the Mann–Whitney test for pairwise comparisons. (*) indicates *P *< 0.05, (***) *P* < 0.001, and (ρ) indicates a trend towards significant differences among infected groups in each tissue.

Parasite DNA in foetal brains was detected in 81.8% of samples in G1 (27/33), 91.7% in G2 (22/24), 66.7% in G3 (26/39), 90.5% in G4 (19/21) and 87.5% in G5 (21/24) (Table 2). Concerning challenge doses, significantly lower parasite detection was found in G3 compared to that in G2 (*P *< 0.05), and a trend towards significance was found compared to those in G1 (*P *= 0.18) and G4 (*P *=0.06). Concerning routes of administration, no significant differences in parasite detection in the foetal brain were found between G2 and G5. Additionally, significantly lower parasite detection was found in aborted foetuses in G3 (12/18) than in aborted foetuses in G2 (20/21) (*P* < 0.05), and a trend towards significance was found compared to that in G4 (12/12) (*P* = 0.05). Likewise, aborted foetuses from G1 (27/33) show lower parasite detection compared to aborted foetuses from G4 (12/12) (*P* < 0.05). However, no significant differences in parasite detection were found among lambs born in any of the infected groups or among aborted foetuses or lambs born in each group. Parasite DNA was not detected in one aborted foetus in G1, in two aborted foetuses belonging to different dams in G3 (these three aborted foetuses were from twin pregnancies with foetal death and parasite detection in only one of the foetuses) or in one lamb in G3 (from a quadruplet pregnancy with parasite detection in three of the four lambs). Furthermore, when different challenge doses were compared, the parasite burden in the foetal brain was lower in G2 (*P *< 0.05) and G3 (*P *< 0.001) compared to that in G1 and G4. Likewise, G2 had a higher parasite load in the foetal brain compared to G3, with a trend towards significance (*P *= 0.06) (Figure 7A). Nevertheless, when routes of administration were compared, no significant differences between parasite load in the foetal brain were found between G2 and G5 (Figure 7B) (Table 2). In addition, a lower parasite load in the foetal brain from aborted foetuses in G2 (G1, *P *< 0.05; G4, *P *< 0.01) and G3 (G1, *P *< 0.05; G4*, P *< 0.01) was found compared to that in G1 and G4. Additionally, a higher parasite load in the foetal brain from lambs born in G4 was found compared to that in G3, with a trend towards significance (*P *= 0.13). However, no significant differences in parasite load in the foetal brain were found between aborted foetuses or lambs in each group. As expected, all placental and foetal samples from G6 were negative.

## Discussion

Ruminant challenge models are critical to the evaluation of vaccine and drug candidates to help tackle ruminant neosporosis and to study pathogenesis and host responses to infection [14]. As an experimental animal model, sheep exhibit several advantages over cattle in terms of cost, space and infrastructure required, ease of handling of the animals and shorter duration of gestation. The pathogenesis of ovine neosporosis is not well known, and in contrast to the clinical outcome in cattle, infection during mid-pregnancy in sheep leads to abortion in most animals [7–9, 28]. The impact of dose and route of challenge in abortion and vertical transmission of *N. caninum* in pregnant sheep has been studied little so far, and the results are difficult to compare. Recently, infection of 90-day-pregnant sheep with 10^6^ tachyzoites of the Nc-Spain7 isolate caused abortion in all of them [8, 23]. Therefore, the aim of this study was to test different challenge doses and routes of administration in ewes infected at mid-term of gestation using the Nc-Spain7 isolate and establish an exogenous transplacental transmission model for ovine neosporosis that mimics natural *N. caninum* infections.

How the dose of tachyzoites equates to the ingestion of sporulated oocysts from definitive hosts and what level of environmental contamination is required to produce a similar outcome through natural exposure is not known [9]. It is possible that the doses of tachyzoites used in previous experiments (even up to 10^8^) have been excessively aggressive for the infected sheep. This suggestion is supported by the fact that previous studies employing lower doses obtained more variable outcomes, ranging from a few aborted foetuses to the birth of weak or healthy lambs [7, 9]. Since intravenous infection of pregnant sheep using 10^6^ tachyzoites of the Nc-Spain7 isolate at mid-gestation resulted in 100% abortion and parasite detection in the foetal brain in 83% of aborted foetuses [8, 23], the challenge doses tested intravenously in the present study were less than the 10^6^ tachyzoites previously assayed; tachyzoites were diluted 1:10 to a minimum concentration of 10^2^ tachyzoites, similar to those evaluated by [9]. Subcutaneous inoculation closely mimics a natural primary infection as the parasite is “processed” through a draining lymph node before circulating in the blood [29]. In cattle, it was reported that subcutaneous infection resulted in a foetal mortality that was 50% reduced compared to intravenous infection [13]. Hence, in this study we investigated the outcome of *N. caninum* infection after subcutaneous inoculation in pregnant ewes using one of the intravenously tested doses. The dose of 10^4^ tachyzoites was chosen for the subcutaneous administration since it is an intermediate-to-high dose between those tested by the intravenous route and also, because in a previous study, intravenous inoculation of 5 × 10^3^ tachyzoites of New Zealand isolates at mid-gestation resulted in abortion in 50% of the ewes [9].

With regard to clinical observations, foetal viability is the most significant parameter to be evaluated in an abortion model of neosporosis [14]. Infection of cattle at mid-term gestation with 10^7^ tachyzoites of the Nc-Spain7 isolate caused foetal death in 50% of infected animals after an experimental period of 6 weeks [19]. All pregnant ewes intravenously infected with 10^5^ tachyzoites (G1) aborted in the same way as pregnant ewes intravenously infected with a 10-fold higher dose (10^6^ tachyzoites) [8, 23]. The intravenous dose causing abortion in 50% of the infected animals was 10^2^ tachyzoites (G4), and the foetal survival rate was higher compared to G1 (10^5^, IV) with a trend towards significance possibly due to the lower number of animals in this group. It is remarkably lower than the intravenous dose of 5 × 10^3^ causing abortion in 50% of the infected animals in the dose-titration study at mid-gestation using Nc-NZ1, Nc-NZ2 and Nc-NZ3 isolates [9], suggesting higher virulence of Nc-Spain7 than New Zealand isolates in pregnant sheep. Likewise, intravenous infection at mid-gestation with 10^5^ tachyzoites of the Nc-Spain7 isolate (G1) resulted in abortion of all pregnant ewes, whereas intravenous infection of pregnant ewes at mid-gestation with 1.7 × 10^5^ tachyzoites of a mixture of the Nc-2 and Nc-Liverpool isolates caused abortion in 67% of the pregnant ewes [7]. However, because [7] and [9] used a mixture of different isolates within the same inoculum to assure infection, it is difficult to establish comparisons with these studies [14]. The median number of abortion days in our study was similar to the time range of abortions in previous *N. caninum* experimental infections in pregnant sheep at mid-gestation [7, 8, 11, 28]. As reported by [9] regarding the differences in the average time between abortion and parturition found after infection with decreasing doses, in the present study, the median survival times in G3 (10^3^, IV) and more markedly in G4 (10^2^, IV) were prolonged compared to those in G1 (10^5^, IV), G2 (10^4^, IV) and G5 (10^4^, SC). In all aborting dams from G3 (10^3^, IV) and G4 (10^2^, IV), the coexistence at the time of euthanasia of live foetuses and dead foetuses in twin pregnancies suggests lower foetal damage in these groups because this observation was only found in one aborting dam in G1 (10^5^, IV) and one aborting dam in G5 (10^4^, SC). Mummified foetuses found in G1 (10^5^, IV) and G2 (10^4^, IV) have already been described after *N. caninum* experimental infection at mid-gestation in pregnant sheep [30]. In G3 (10^3^, IV), G4 (10^2^, IV) and G5 (10^4^, SC), pregnant ewes gave birth prematurely, similar to some pregnant ewes infected with 5 × 10^3^ *N. caninum* tachyzoites [9]. Consequently, in G3 (10^3^, IV), G4 (10^2^, IV) and G5 (10^4^, SC), stillborns and lambs that died soon after birth show a significant decrease in their bodyweight, as previously described [28]. Likewise, a more significant decrease in lamb weight in G3 (10^3^, IV) was found because it is known that with increasing litter size, the weight of the lambs is lower [31]. The presence of a large number of stillborn lambs and weak lambs dying within 24 h after birth could be explained by the absence of differences in parasite detection, parasite load and lesion severity from the foetal brain between aborted foetuses and lambs that gave birth in each group.

Another clinical parameter associated with infection is rectal temperature, and its increase is probably associated with the first replication cycles of the parasite in tissues and organs [14]. Moreover, different temperature responses have been associated with the dose of parasite inoculums [9, 30, 32, 33]. Intravenously challenged groups show a unique fever peak after infection, although a dose-dependent delay in the time of rectal temperature increase was found compared to the infection with 10^6^ tachyzoites of the Nc-Spain7 isolate [23], suggesting delayed parasite replication as lower infection doses were applied. However, as reported with 10^6^ tachyzoites of the Nc-Spain7 isolate [23], G1 (10^5^, IV), G2 (10^4^, IV) show maximum rectal temperature on day 7 pi. Whereas the increase in rectal temperature persisted for 4 days in G1 (10^5^, IV), G2 (10^4^, IV) and G3 (10^3^, IV), a less prolonged period with 3 days of rectal temperature increase was found in G4 (10^2^, IV). Similar to different temperature responses found between intravenous and subcutaneous *N. caninum* challenge in cattle [13], after subcutaneous challenge in G5 (10^4^, SC), a lower rectal temperature increase was found compared to intravenous challenge in G2 (10^4^, IV). Likewise, a similar temperature response was found in G5 (10^4^, SC) compared to the infection in sheep with the same dose (10^4^ tachyzoites) of the Nc-Liverpool isolate [30]. The biphasic temperature response found in the subcutaneous challenge group (G5) had been previously described after subcutaneous challenge in sheep [11, 28, 30] and cattle [33, 34]. Previous studies in cattle have described differences in rectal temperatures between aborting and non-aborting dams [19, 35] in the same way as observed in G4 (10^2^, IV) in the present study.

Prefemoral lymph nodes were chosen as inoculation sites in G5 (10^4^, SC) because they have been widely used for subcutaneous *N. caninum* challenge in cattle [12, 13, 32–34, 36] and sheep [11, 28, 30]. The clinical evaluation in G5 (10^4^, SC) revealed enlargement of the left prefemoral lymph node as previously described [13, 32, 33, 37] after subcutaneous *N. caninum* challenge in cattle.

Intracellular protozoan parasites usually induce and are controlled by cellular immune responses. IFNγ plays a relevant role in controlling early *N. caninum* dissemination [38, 39] and protecting against abortion in naturally infected cows [40]. Very short-lived IFNγ levels were produced in antigen-specific stimulation analyses at the end of the first and during the second week following infection with *N. caninum* and prior to mounting a specific IgG response. Similar IFNγ kinetics have been described in previous reports carried out in cattle upon stimulation of peripheral blood mononuclear cells (PBMCs) [18] or in sheep serum [41]. In G1 (10^5^, IV), IFNγ released upon stimulation increased on day 7 pi in the same way that intravenous infection with 10^6^ tachyzoites of the Nc-Spain7 isolate [23]. Nevertheless, the time course of IFNγ shows a delay until day 10 pi for IFNγ release after intravenous challenge in G2 (10^4^, IV) and G3 (10^3^, IV) and subcutaneous challenge in G5 (10^4^, SC), although large individual variations were observed as previously described [42]. Likewise, no significant increase in IFNγ was observed in G4 (10^2^, IV) compared to the uninfected group (G6), which might have led to lower initial control of parasitaemia at the peripheral level, allowing a higher number of parasites to reach the placenta [38, 43]. In fact, no differences in parasite load in the foetal brain between G4 (10^2^, IV) and G1 (10^5^, IV) could be due to the absence of a significant increase in IFNγ levels in pregnant ewes from G4 (10^2^, IV) because a threshold IFNγ response is required to be beneficial against *N. caninum* [44, 45]. Recently, it has been shown that the immune response appears to lead to superior priming of a cell-mediated immune response in dams carrying live foetuses *versus* dams carrying dead foetuses [46, 47]. In this way, ewes that gave birth in G2 (10^4^, IV) had higher IFNγ levels on day 10 pi than those that aborted. Although no significant differences were found in IFNγ levels in G4 (10^2^, IV) compared to the uninfected group (G6), a delay in the IFNγ peak was detected in ewes that gave birth. That, along with differences in rectal temperatures between aborting ewes and ewes that gave birth could suggest decreased early-stage replication of the parasite in ewes that gave birth in G4 (10^2^, IV).

After intravenous infection of pregnant sheep at mid-gestation with 10^6^ tachyzoites of the Nc-Spain7 isolate, IgG levels increased from day 12–14 pi [23, 41], whereas IgG levels increased from day 21 pi in G1 (10^5^, IV). Furthermore, G1 (10^5^, IV) exhibited higher IgG levels than those found with lower doses, possibly due to exposure to more abundant antigen and increased lymphoid stimulation similar to that reported by [9, 30, 32, 48]. In this study, all challenged animals show seroconversion by ELISA, whereas the lower dose tested (50 tachyzoites of Nc-NZ1, Nc-NZ2 and Nc-NZ3 isolates) by [9] revealed one seronegative animal at parturition by IFAT.

Concerning offspring, all aborted foetuses and lambs from the infected groups had seropositive IFAT titres with no significant differences between challenge doses or routes of administration. Altogether, this finding indicates that once infection is established, it cannot be cleared from the host, and vertical transmission of the parasite occurred in all infected animals. In contrast, in a previous dose-titration study, none of the lambs and only 3 out of 5 lambs born from pregnant ewes intravenously infected with 50 and 5 × 10^3^ *N. caninum* tachyzoites (Nc-NZ1, Nc-NZ2 and Nc-NZ3 isolates), respectively, were seropositive by IFAT [9].

Because immune responses are not accurate enough to be used as indicators for disease or protection [14], parasite detection and quantification and histopathological assessment are essential. All placentomes from infected ewes were PCR positive, and no significant differences in parasite load or lesion severity were found, so immune responses were unsuccessful in preventing the colonization and multiplication of *N. caninum* in the placentomes of aborting ewes. No difference was found in parasite detection between placentomes from intravenously infected ewes at mid-gestation with 10^6^ tachyzoites of the Nc-Spain7 isolate (PCR-positive samples from 83 to 100%) [8, 23] and placentomes from intravenously infected aborting ewes in this study. *N. caninum* DNA was also widely detected in cotyledons from ewes that gave birth with no significant differences in parasite detection, similar to that reported by [9], which found all PCR-positive cotyledons in ewes infected with 5 × 10^3^ *N. caninum* tachyzoites. However, lower parasite loads were found in cotyledons from ewes that gave birth in G3 (10^3^, IV), which, along with lower IFNγ levels detected, could suggest mild infection in these animals. When routes of administration were compared, no significant differences were identified in parasite detection, parasite load or lesion severity in placentomes, however, cotyledons from the subcutaneously infected group (G5) show lower parasite burden compared to G2 (10^4^, IV) with a trend towards significance, maybe influenced by the lower number of animals in this group.

In transplacental transmission models for ruminant neosporosis, it is crucial to evaluate parasite presence, parasite load and lesions in foetal tissues [14]. The central nervous system has been described as the target tissue in foetuses from *N. caninum* infection of cattle [19] and sheep [8] at mid-pregnancy. Concerning infective doses, there were no significant differences among detection percentages in foetal brains from G1 (10^5^, IV), G2 (10^4^, IV) and G4 (10^2^, IV), nor when they were compared to detection in foetal brains after intravenous infection of pregnant ewes with 10^6^ tachyzoites of the Nc-Spain7 isolate at mid-gestation (94% of PCR-positive samples) [23]. These results were not in accordance with differences in the proportion of positive brains in foetuses/lambs of a dose-titration study in pregnant sheep at mid-gestation using other isolates [9]. Conversely, the lower detection percentage in foetal brains from G3 (10^3^, IV) could be because of the higher number of foetuses per dam in this group; fewer parasites that cross the placental barrier reach each foetus. Brain-negative foetuses arising from multiple pregnancies have already been reported in ewes infected at mid-gestation with 10^6^ tachyzoites of Nc-Spain7 [8]. Foetuses and lambs showing PCR-negative brains in this study were seropositive by IFAT in the same way as in [16], and brain lesions were identified, suggesting the presence of very low parasite load in their brains.

We hereby describe the outcome of *N. caninum* infection in pregnant sheep at mid-gestation by performing experimental infections using different numbers of tachyzoites of the virulent Nc-Spain7 isolate and different routes of inoculation. Intravenous infection with 10^5^ tachyzoites was sufficient to trigger 100% abortion in the same way as 10^6^ tachyzoites previously assayed. In addition, intravenous infection with 10^5^ tachyzoites shows distinct immune responses and parasite load in the foetal brain. Surprisingly, the differences between the highest and the lowest intravenous doses were much smaller than expected, and we here demonstrate that experimental infection with as few as 100 tachyzoites could induce abortion in 50% of the ewes, and parasite load in the foetal brain was similar to that with the highest dose. Regarding the routes of inoculation, subcutaneous infection with 10^4^ tachyzoites shows similar abortion rates and vertical transmission to intravenous infection.

In conclusion, with the doses and routes of administration evaluated, we propose that future studies using an abortion model for ovine neosporosis should be carried out using the intravenous route of administration and a challenge dose of 10^5^ tachyzoites (100% abortion and vertical transmission), which will then allow obtaining more accurate and realistic conclusions in studies testing vaccine and drug candidates. However, further studies are necessary to evaluate the outcome of infection with 10^5^ tachyzoites by the subcutaneous route of administration.
